# Alpha 1 Antitrypsin Inhibits Dendritic Cell Activation and Attenuates Nephritis in a Mouse Model of Lupus

**DOI:** 10.1371/journal.pone.0156583

**Published:** 2016-05-27

**Authors:** Ahmed S. Elshikha, Yuanqing Lu, Mong-Jen Chen, Mohammad Akbar, Leilani Zeumer, Andrea Ritter, Hanaa Elghamry, Mahmoud A. Mahdi, Laurence Morel, Sihong Song

**Affiliations:** 1 Department of Pharmaceutics, College of Pharmacy, University of Florida, Gainesville, Florida, United States of America; 2 Department of Pathology, Immunology, and Laboratory Medicine, University of Florida, Gainesville, Florida, United States of America; 3 Department of Pharmaceutics, Zagazig University, Zagazig, Sharkia, Egypt; Instituto Nacional de Ciencias Medicas y Nutricion Salvador Zubiran, MEXICO

## Abstract

Systemic lupus erythematosus (SLE) is an autoimmune disorder with a worldwide distribution and considerable mortality and morbidity. Although the pathogenesis of this disease remains elusive, over-reactive dendritic cells (DCs) play a critical role in the disease development. It has been shown that human alpha-1 antitrypsin (hAAT) has protective effects in type 1 diabetes and rheumatoid arthritis mouse models. In the present study, we tested the effect of AAT on DC differentiation and functions, as well as its protective effect in a lupus-prone mouse model. We showed that hAAT treatment significantly inhibited LPS (TLR4 agonist) and CpG (TLR9 agonist) -induced bone-marrow (BM)-derived conventional and plasmacytoid DC (cDC and pDC) activation and reduced the production of inflammatory cytokines including IFN-I, TNF-α and IL-1β. In MRL/lpr mice, hAAT treatment significantly reduced BM-derived DC differentiation, serum autoantibody levels, and importantly attenuated renal pathology. Our results for the first time demonstrate that hAAT inhibits DC activation and function, and it also attenuates autoimmunity and renal damage in the MRL/lpr lupus model. These results imply that hAAT has a therapeutic potential for the treatment of SLE in humans.

## Introduction

Systemic lupus erythematosus (SLE) is a prototypic autoimmune disease characterized by dysregulation in multiple arms of the immune system and the production of hallmark autoantibodies [1]. Increasing evidence indicates that dendritic cells (DCs) play critical roles in the development of SLE [2,3,4,5,6,7,8,9]. DCs are activated by immune complexes and act as a bridge between the innate and adaptive immune responses [10]. DCs express Fc receptors, through which immune complexes can bind and internalize [11]. Immune complexes formed by autoantibodies against nucleic acid/protein complexes stimulate endosomal Toll-like receptors (TLRs) [11]. This stimulation leads to DC activation and cytokine production, which result in the activation of T and B cells [11]. Moreover, mice deficient in either conventional DCs [cDCs] or plasmacytoid DCs [pDCs] are protected from developing lupus nephritis [9].

MRL/lpr mice are a classical spontaneous model of lupus, in which multiple organs are affected and glomerulonephritis is the major cause of mortality [12]. Deleting DCs ameliorated disease in MRL/lpr mice by limiting the expansion of inflammatory T cells as well as the number of autoantibody producing cells [9]. The role and requirement of DCs in the NZM2410-derived B6.TC model of lupus [13] has not been directly tested by depletion. However, several studies have shown that they are likely to contribute to disease. Stimulated DCs from B6.TC mice enhanced B cell proliferation and antibody production [7], and prevented regulatory T cells induction [6]. The adoptive transfer of DCs bearing the *Sle3* susceptibility locus into non-autoimmune C57BL/6 mice led to marked production of anti-nuclear antibodies (ANA) when coupled with LPS co-administration [14]. Finally, B6.TC lupus-prone mice express a gene profile with interferon signature genes and DCs are important source for type I interferon [4].

Several studies have shown that DCs from SLE patients display an increased expression of the co-stimulatory molecules CD40 and CD86 and a higher ratio of activating to inhibitory Fc gamma receptors when compared to DCs from healthy persons, suggesting that DC maturation may participate in an inefficient peripheral tolerance in these patients [15]. Moreover, abnormal co-stimulatory profiles have been reported in DCs from (NZB x NZW) F1, NZM2410, and B6.TC lupus-prone mice [6,16]. Because of the pivotal role of DCs in lupus, including abnormal activation, ability to induce autoimmunity and to produce large amounts of IFNα/β [8,9,16,17,18], targeting DCs has therapeutic potential for the prevention or treatment of lupus.

Innate immune sensing by TLRs is a major activation pathway for DCs. Over-expression of TLR4 in mice leads to the production of anti-dsDNA IgG and immune complex-mediated glomerulonephritis, suggesting that TLR4 signaling plays a role in lupus progression [19]. Endogenous DNA is recognized by TLR9 leading to activation of pDCs and production of type I interferon [20], which is believed to play a crucial role in SLE pathogenesis [21]. In addition, type I IFN produced by pDCs lowers the activation threshold for TLR agonists on cDCs [9].

Human alpha-1 antitrypsin (hAAT) is a multifunctional protein with anti-inflammatory, cytoprotective and immunoregulatory properties. hAAT protected islet cell allograft from rejection [22], blocked β cell apoptosis [23], prevented pulmonary emphysema [24], and inhibited angiogenesis and tumor growth [25]. hAAT inhibited LPS-stimulated release of TNF-α and IL-1β, and enhanced the production of anti-inflammatory IL-10 in monocytes [26]. We have previously shown that hAAT gene and protein therapy prevented and reversed type 1 diabetes in NOD mice [27,28,29], and delayed collagen induced arthritis in DBA/1 mice [30,31]. Based on these results, we hypothesize that hAAT may hold therapeutic potentials in controlling DC functions and the development of SLE. In the present study, we tested the effect of hAAT on DC differentiation, maturation, and expression of cytokines *in vitro* and *in vivo* as well as its effect on autoantibodies production and nephritis development in MRL/lpr mice.

## Materials and Methods

### Mice

Female or male C57BL/6 (B6) and female MRL/lpr mice were purchased from the Jackson Laboratory. All mice were maintained in specific pathogen-free conditions and monitored daily. At 7 weeks of age, MRL/lpr mice were randomly distributed into two groups receiving either 100μl PBS or 2 mg clinical grade human AAT (Prolastin C^®^, Grifols, Inc., NC) in 100 μl of PBS injected intra-peritoneally (i.p.) every 3 days. The primary endpoint of the experiment was 11 weeks of treatment (at 18 weeks of age). If an animal developed severe disease, or showed two successive >300 mg/dl proteinuria scores or a body weight reduction >15% within a week, then the animal was sacrificed. One out of 10 PBS-treated MRL/lpr mice was sacrificed before the 11-week endpoint of the experiment because of high proteinuria, while all of the hAAT-treated mice reached the 11-week endpoint. All mice were sacrificed by cervical dislocation under anesthesia. All experiments were conducted according to protocols approved by the University of Florida Institutional Animal Care and Use Committee.

### Ethic statement

Experiments using mice reported here were approved by the Institutional Animal Care and Use Committee of the University of Florida (UF #201307848).

### Conventional DCs preparation and cell cultures

Bone-marrow-derived dendritic cells (BMDCs) were obtained from 2–3 months old B6 or MRL/lpr mice as previously described [32] with some modifications. Briefly, BM single cell suspensions were isolated from femurs and tibias, and depleted of red blood cells (RBCs) with a RBC lysis buffer solution (Stem cell technologies). Cells were cultured with or without 1 mg/ml hAAT for 5 days in RPMI 1640 medium (Corning Cellgro) supplemented with 10% heat inactivated fetal bovine serum (Thermo Scientific), 100 U/ml penicillin/streptomycin (Corning Cellgro), 10 ng/ml recombinant murine GM-CSF and 5 ng/ml IL-4 (R&D systems). On day 3, half of the medium was replaced with fresh medium supplemented with hAAT, GM-CSF and IL-4. On day 4, cells were stimulated by adding or not LPS (0.5μg/ml, Sigma) or CpG-ODN 1826 (10μg/ml, InvivoGen) for an additional 24 hrs. The supernatants were used to detect cytokines and cells were stained for surface markers and analyzed by flow cytometry. Similarly, BMDCs were obtained from PBS and hAAT- treated MRL/lpr mice at week 11 after treatment. Cells were stimulated with LPS at day 4 of DC induction without hAAT treatment. All BMDCs were collected at day five and analyzed by flow cytometry and supernatant stored at -80°C for cytokine detection.

### Plasmacytoid dendritic cells induction

BM cells were differentiated into pDC in medium containing Flt3L [33]. Briefly, BM cells were plated at 2 X 10^6^ cells/well in RPMI 1640 medium supplemented with 10% heat inactivated fetal bovine serum, 100 U/ml penicillin/streptomycin and 200 ng/ml Flt3L (R&D systems). During the induction, cells were treated with or without AAT (0.6 or 1mg/ml). At day 4, half of the medium was replaced with fresh medium supplemented with hAAT and Flt3L. On day 8, cells were stimulated with or without CpG-ODN 1826 for an additional 24 hr. Then, the supernatants were used for detection of cytokines and mouse AAT (mAAT), and cells were analyzed by flow cytometry.

### Flow cytometry

Briefly, cells were first blocked on ice with staining buffer (PBS, 5% horse serum, 0.09% sodium azide) supplemented with 10% rabbit serum and pretreated with anti-CD16/CD32 (2.4 G.2) to block FcR-mediated binding. Cells were then stained with the following: FITC-, PE-, or biotin-conjugated antibodies, CCR9 (CW-1.2), CD11b (M1/70), CD11c (HL3), CD40 (3/23), CD80 (16/10 A1), CD86 (GL1), PDCA-1 (927), I-A^b^ (AF6-120.1), and I-A/I-E (M5/114.15.2). All antibodies were obtained from BD Biosciences or eBiosciences. Biotin-conjugated Abs were revealed using streptavidin-PERCP-Cy 5.5 (BD Biosciences). Cell staining was analyzed using a FACSCalibur (BD Biosciences).

### Cytokine assays

IL-6, TNF-α, and IL-1β in cell culture medium and serum samples were detected by ELISA (PeproTech). BAFF levels in culture medium and serum samples were measured by ELISA (R&D systems). Type I IFN (IFN-I) was quantified using murine IFN-I sensor B16-Blue^™^ IFN-α/β cells (InvivoGen). Briefly, 20 μl of culture media were combined to 180 μl of medium containing B16-Blue^™^ IFN α/β cells (7.5 x 10^4^) in each well of a 96-well plate. The plate was incubated at 37°C in 5% CO_2_ for 22-24h. On the second day, 180 μl of QUANTI-Blue^™^ was combined to 20 μl of induced B16-Blue^™^ IFN α/β cells supernatant in a flat-bottom 96-well plate, which was incubated at 37°C for 3–4 h. The levels of purple/blue color were detected at 630 nm.

### Detection of serum hAAT concentration and anti-AAT antibody

To detect the hAAT level in the mouse serum, a hAAT specific ELISA was performed as previously described [34]. To assess if hAAT would induce generation of anti-hAAT antibodies in mice post- hAAT administration, the serum level of anti-hAAT antibody was determined weekly by using anti-hAAT antibody ELISA as previously described [34].

### Detection of mouse AAT by ELISA

Mouse AAT levels in lupus mouse serum and pDC culture medium were detected by ELISA. Briefly, pooled B6 (adult male) mouse serum was used as a standard, in which mouse AAT concentration was defined as a one relative unit. Samples (culture medium or sera from lupus mice) and standards were diluted and incubated in a microtiter plate (Immulon 4, Dynex Technologies) in Voller’s buffer overnight at 4°C. Plates were blocked with 3% bovine serum albumin (Sigma) for 1 h at 37°C. Then samples were incubated for 1 h at 37°C. Chicken anti-mouse alpha 1-antitrypsin polyclonal antibody (1:1600 dilution, MyBioscience) and HRP-conjugated goat anti-chicken-IgG antibody (1:5000 dilution, ThermoScientific) were added and incubated for 1 h at 37°C. The plates were washed with PBS-Tween 20 between reactions. After adding substrate (O-Phenyldiamine, Sigma), plates were read at 490 nm on an MRX microplate reader (Dynex Technologies). The OD reading of each sample was used to calculate relative unit based on the standard curve.

### Evaluation of glomerulonephritis

Proteinuria levels were determined by a semi-quantitative method with Albustix strips (Siemens), using a 0–4 scale (0: negative, 1: 30 mg/dl, 2: 100 mg/dl, 3: 300 mg/dl and 4: over 2000 mg/dl of urinary protein). Urinary albumin excretion was determined by ELISA (Kamiya Biomedical). Kidneys were fixed in 10% buffered formalin, embedded in paraffin, and sections were stained with Periodic acid-Schiff (PAS). The average of area and number of glomeruli (25–30 glomeruli per slide) were measured with image analysis software (Aperio Imagescope^®^).

### Gross pathology

Lymphadenopathy (cervical, brachial, and inguinal) was scored by palpation using a previously reported 0–3 scale (0 = none; 1 = small, at one site; 2 = moderate, at 2 sides; 3 = large, at three or more sites) [35]. Skin lesions were scored using a 0–3 grade as previously described (0 = none; 1 = mild (snout and ears); 2 = moderate, < 2 cm (snout, ears, and intrerscapular); and 3 = severe, > 2 cm (snout, ears, and intrerscapular)) [35].

### Autoantibody measurement

For the detection of anti-nuclear antibodies (ANAs), serum was diluted 1:40 and used for indirect immunostaining of Hep-2 slides (Inova Diagnostics) with Alexa Flour 488-conjugated goat anti-mouse IgG (BD Biosciences). Slides were fixed with Fluoromount (Sigma-Aldrich) and fluorescence intensity was analyzed using the ImageJ program. Detection of anti-dsDNA IgG was performed as previously described [13]. Briefly, Immulon 2 HB plates (Thermo Scientific) pre-coated with 1 mg/ml methylated BSA (Sigma) in PBS, were coated with 50 μg/ml of dsDNA, washed with PBS and blocked with 0.1% gelatin containing 3% BSA/3mM EDTA. The dsDNA was reacted with diluted serum (1:2400 dilutions in 0.1% gelatin containing 2% BSA, 3mM EDTA and 0.05% Tween 20). HRP-conjugated goat anti-mouse IgG (Southern Biotechnology) was used as secondary antibody at a 1:1000 dilution. The HRP activity was detected using pNPP substrate. ELISA data was normalized to a high titer B6.TC mouse given an arbitrary level of 100 units and run in serial dilution on each plate.

### Statistical analysis

Statistical analysis was performed with GraphPad Prism software package V5.04 (La Jolla, CA, USA) with the tests indicated in the text. Data were subjected to analysis of variance (ANOVA) with Tukey’s post-hoc test, two-tailed Student’s *t* test, or Mann-Whitney test. Graphs show mean and standard error of the mean (SEM), and statistical significance is presented as *P< 0.05, **P <0.01, ***P<0.001.

## Results

### Human AAT inhibited cDC maturation and cytokine production

**Fig 1:**
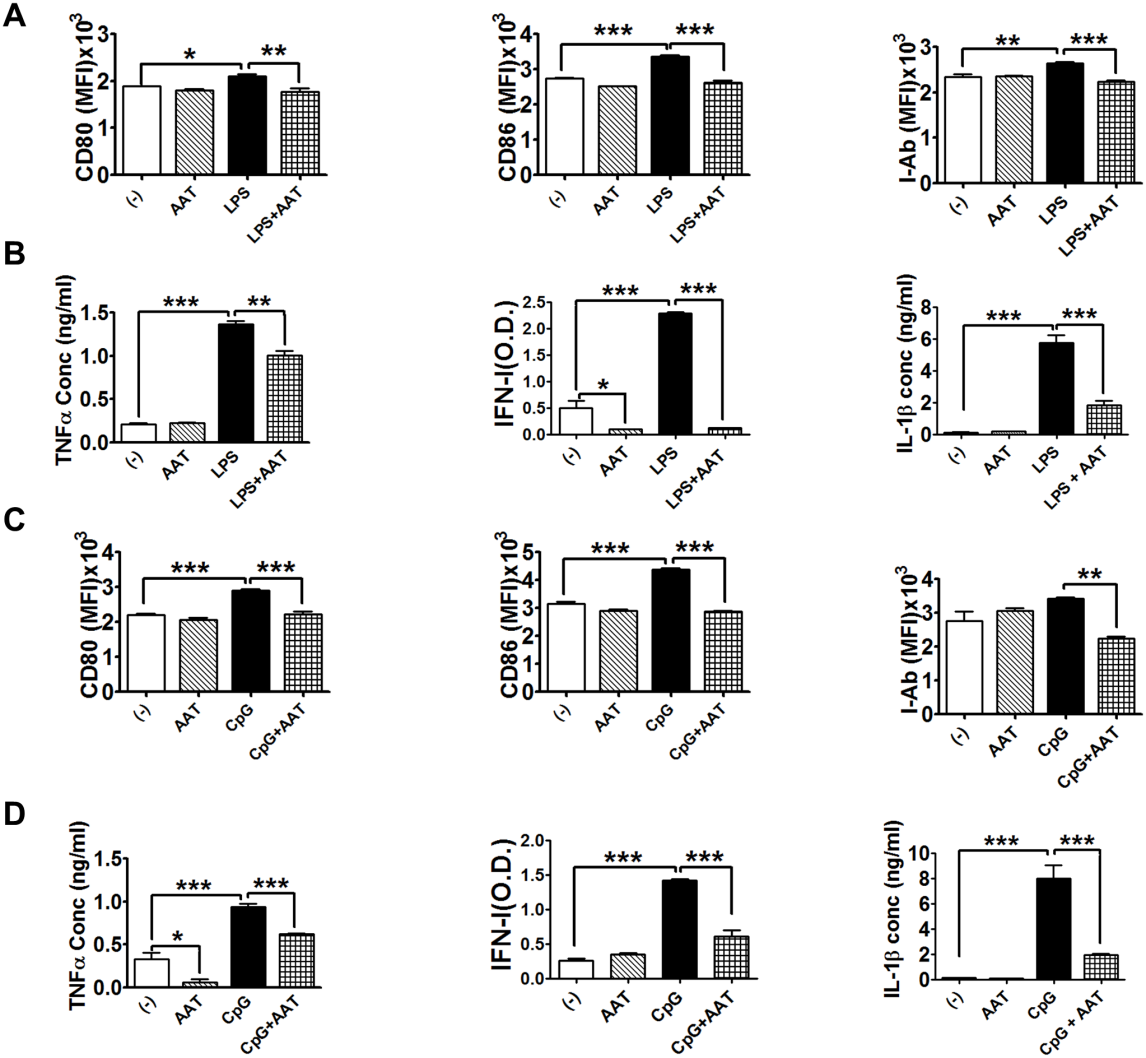
Human AAT inhibited cDC maturation and cytokine secretion. BM cDCs from B6 mice were generated in vitro in the presence of GM-CSF and IL-4 for 4 days with or without hAAT and then stimulated with 0.5 μg/ml LPS or 10μg/ml CpG for an additional 24 hr prior to FACS analysis. (A) CD80, CD86 and I-A^b^ expression (measured as mean fluorescence intensity, MFI) in B6 DCs stimulated with LPS. (B) TNF-α, IFNI, and IL-1β secretions in supernatants of B6 DCs stimulated with LPS. (C) CD80, CD86 and I-Ab expression in B6 DCs stimulated with CpG. (D) Secretion of TNF-α, IFN-I, IL-1β, and IL-6 by B6 DCs stimulated with CpG. P values of One-Way-ANOVA using Tukey’s post-hoc test are indicated as * P<0.05; ** P<0.01; *** P<0.001, n = 3.

DCs express TLR4 and produce pro-inflammatory cytokines such as IL-1β, IL-6 or TNF-α when activated by TLR4 agonist LPS [36,37]. In order to test the effect of hAAT on cDC differentiation and maturation in response to LPS stimulation, BM cDCs from B6 mice were induced by IL-4 and GM-CSF in the presence or absence of hAAT. At day 4, cells were stimulated with LPS for an additional 24 hrs, and then harvested for the evaluation of cDC differentiation, maturation and cytokine production. While no or minimal effect on cDC differentiation was observed (S1A Fig), hAAT treatment significantly inhibited LPS induced expression of the co-stimulatory molecules (CD80 and CD86) as well as I-A^b^ (Fig 1A) In addition, hAAT treatment significantly inhibited LPS-induced TNF-α, IFN-I and IL-1β production (Fig 1B). These data indicated hAAT attenuated TLR4 mediated cDC maturation in non-autoimmune mice.

TLR9 activation plays an important role in inducing anti-chromatin and anti-DNA autoantibody production in murine lupus [38]. To investigate whether hAAT can inhibit TLR9 agonist (CpG) induced DC maturation, BMDCs from C57BL/6 mice were stimulated at day 4 with CpG for an additional 24 hrs in the presence or absence of hAAT. As shown in S1B Fig, hAAT had no or minimal effect on cDC differentiation (control vs. AAT group) and some enhancing effect on total cDC expansion in the presence of CpG. Importantly, hAAT treatment significantly inhibited CpG induced expression of CD80, CD86 and I-A^b^ (Fig 1C). Moreover, hAAT treatment significantly inhibited CpG induced TNF-α, IFN-I and IL-1β production (Fig 1D). Together, these data demonstrated the inhibitory effect of hAAT on cDC maturation and the cytokine secretion upon stimulation with TLR4 or TLR9 agonists.

### Human AAT inhibits pDC maturation

**Fig 2:**
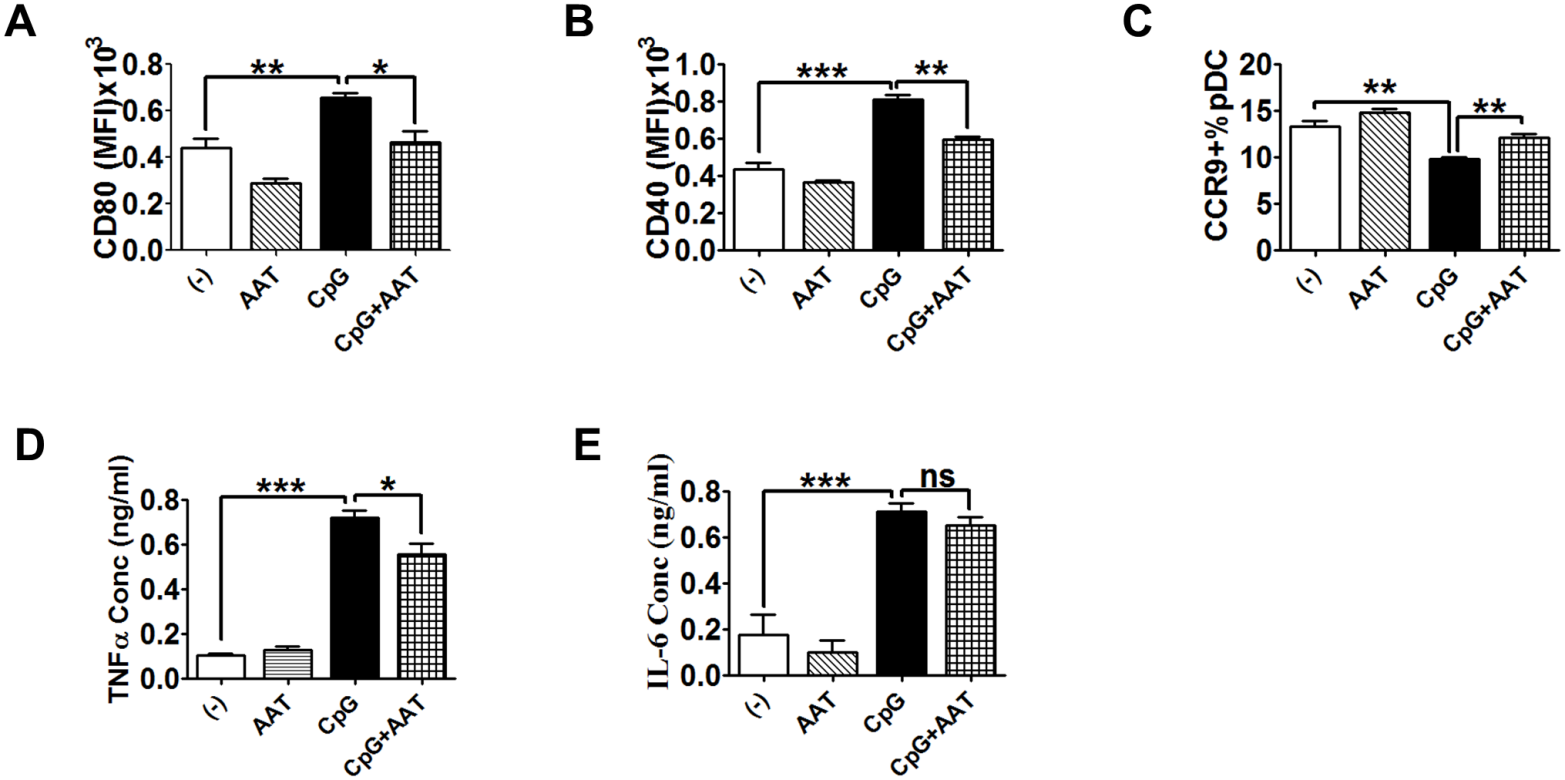
Human AAT inhibited pDC maturation and cytokine secretion. BM pDCs from B6 mice were differentiated with or without hAAT using Flt3L for 8 days then stimulated with 10μg/ml CpG for an additional 24 hr prior to FACS analysis. (A) CD80, (B) CD40, and (C) CCR9 expression. (D) TNF-α and (E) IL-6 levels in pDC culture media. P values of One-Way-ANOVA using Tukey’s post-hoc test are indicated as * P<0.05; ** P<0.01; *** P<0.001, n = 3.

Since pDCs are essential for lupus like disease development in mice [39,40], we next examined the expression of CD40 and CD80 on the surface of pDCs following stimulation with TLR9 agonist (CpG). First, we observed that hAAT attenuated the percentage of PDCA-1^+^CD11c^+^ cells in control and hAAT treated groups while it had no significant effect on CpG stimulated groups (S2 Fig). Consistent with the observation in cDCs (Fig 1), hAAT markedly decreased CD40 and CD80 expression on pDCs upon stimulation with TLR9 agonist (Figs 2A, 2B and S3). As expected [41], CpG treatment reduced CCR9 expression. However, hAAT treatment significantly increased CCR9 expression in B6 pDCs (Fig 2C), again indicating that hAAT inhibited pDC maturation. We also found that levels of TNF-α were significantly reduced (Fig 2D) in hAAT treated groups. Levels of IL-6 (Fig 2E) were also reduced in hAAT-treated pDCs although it did not reach statistical difference. These results indicated that hAAT treatment also inhibited TLR9-mediated pDC maturation. Mouse pDCs produced detectable levels of endogenous AAT (S4A Fig). However CpG had no effect on mouse AAT production (S4B Fig). This suggests that the small amount of endogenous mouse AAT did not contribute to the results observed with exogenous hAAT.

### The endogenous mouse AAT level is decreased in MRL/lpr mice during disease progression

**Fig 3:**
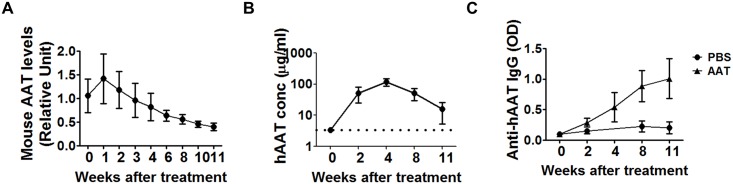
Detection of endogenous mouse AAT, exogenous human AAT, and anti-human AAT neutralizing antibody in MRL/lpr mice (N = 10) by ELISA. (A) Mouse AAT levels (relative unit to C57BL/6) in PBS-treated MRL mice. (B) hAAT levels detected in hAAT injected group. Note: at week 2 and 8, animals were bled at 2 days after hAAT injection; at week 4, animals were bled at 1 day after the injection; at week 11, animals were bled 3 days after the injection. Dashed line is the lower limit of quantification (LLOQ). The serum concentration of human AAT from the PBS-treated group was below LLOQ. (C) Relative levels of anti-human AAT neutralizing antibody in MRL/lpr mice following multiple-dose human AAT administrations in MRL/lpr mice.

To investigate the effect of hAAT in vivo, we employed MRL/lpr mice, which spontaneously develop lupus that is characterized by high levels of auto-antibodies and develop immune complex-type nephritis, lymphadenopathy and splenomegaly [42,43]. Female MRL/lpr mice (7 weeks old) were treated with clinical-grade hAAT or PBS as a control for 11 weeks, and sacrificed for immunological and pathological examinations. In this experiment, we first detected mouse AAT in PBS injected control groups and showed that serum mouse AAT levels were decreased gradually (Fig 3A). We also detected hAAT levels (Fig 3B) and anti-hAAT antibody levels in the hAAT-injected group (Fig 3C).

### Human AAT treatment attenuated BMDC differentiation and maturation in MRL/lpr mice

**Fig 4:**
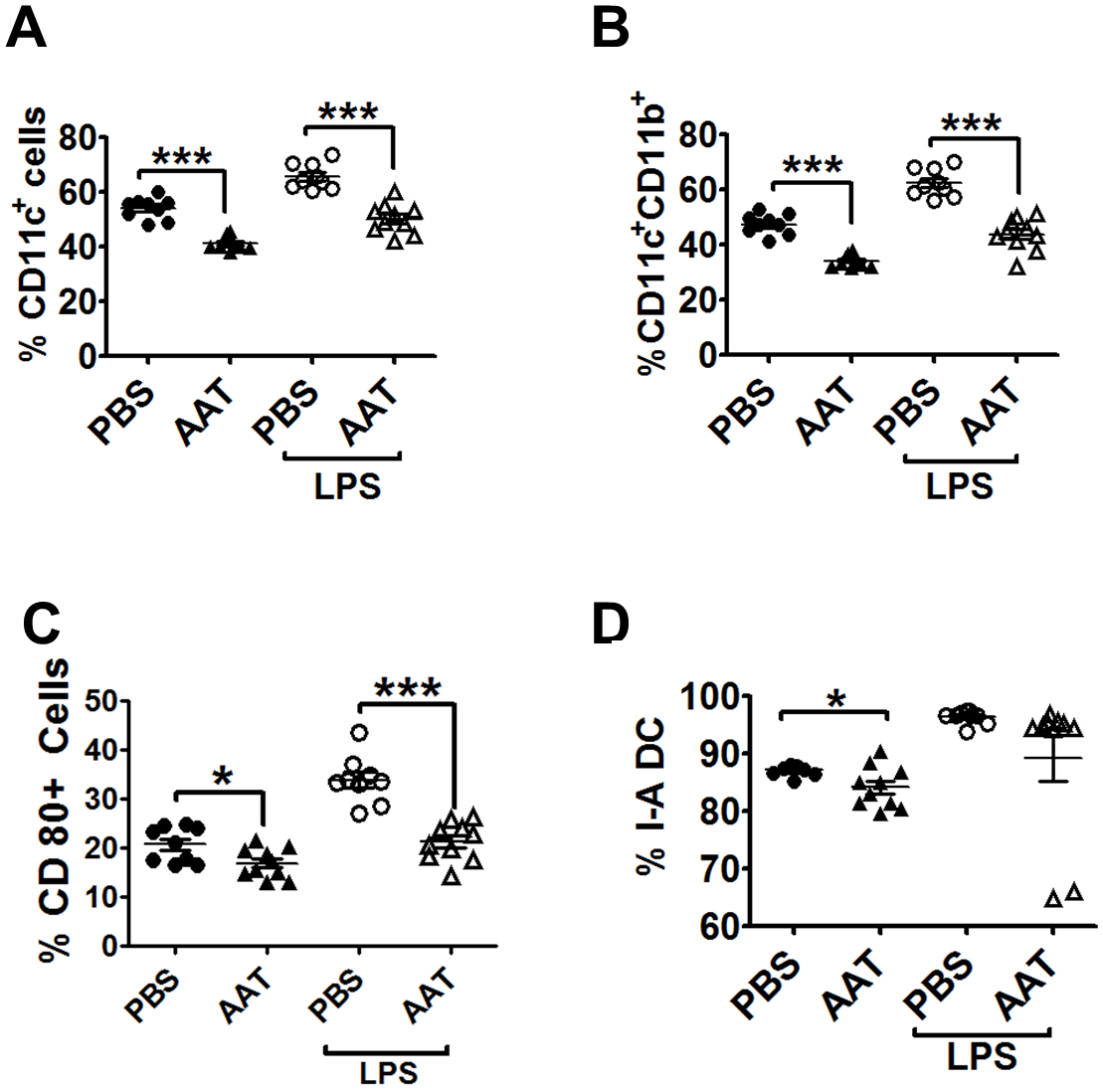
*In vivo* hAAT treatment attenuated BMDCs differentiation and maturation in MRL/lpr mice. BMDCs from MRL/lpr mice treated with hAAT or PBS for 11 weeks were stimulated with LPS for 24 hrs. (A) Percentages of CD11c^+^ and (B) CD11b^+^CD11c^+^. (C-D) Percentages of CD80^+^ (C) and I-A (D) expressing BMDCs. P values of Student’s *t*-test are indicated as * P<0.05; ** P<0.01; *** P<0.001.

When BM cDCs were differentiated from both the control and hAAT groups and analyzed by flow cytometry, the percentage of CD11c^+^ and CD11c^+^CD11b^+^ cells obtained from hAAT-treated mice were significantly lower than that in control mice (Fig 4A and 4B). In addition, the expression of CD80 and I-A in immature cDCs was significantly lower in the AAT-treated group (Fig 4C and 4D). We also observed that the BM cDCs from the hAAT-treated group were less responsive to LPS stimulation than those from the control group (Fig 4A–4D). Consistent with our in vitro data in B6 mice (Figs 1 and 2), these results clearly demonstrated that the hAAT treatment significantly attenuated BM cDC differentiation and maturation stimulated by TLR agonists in the lupus mouse model.

### Human AAT treatment attenuated autoimmune pathology in MRL/lpr lupus mice

**Fig 5:**
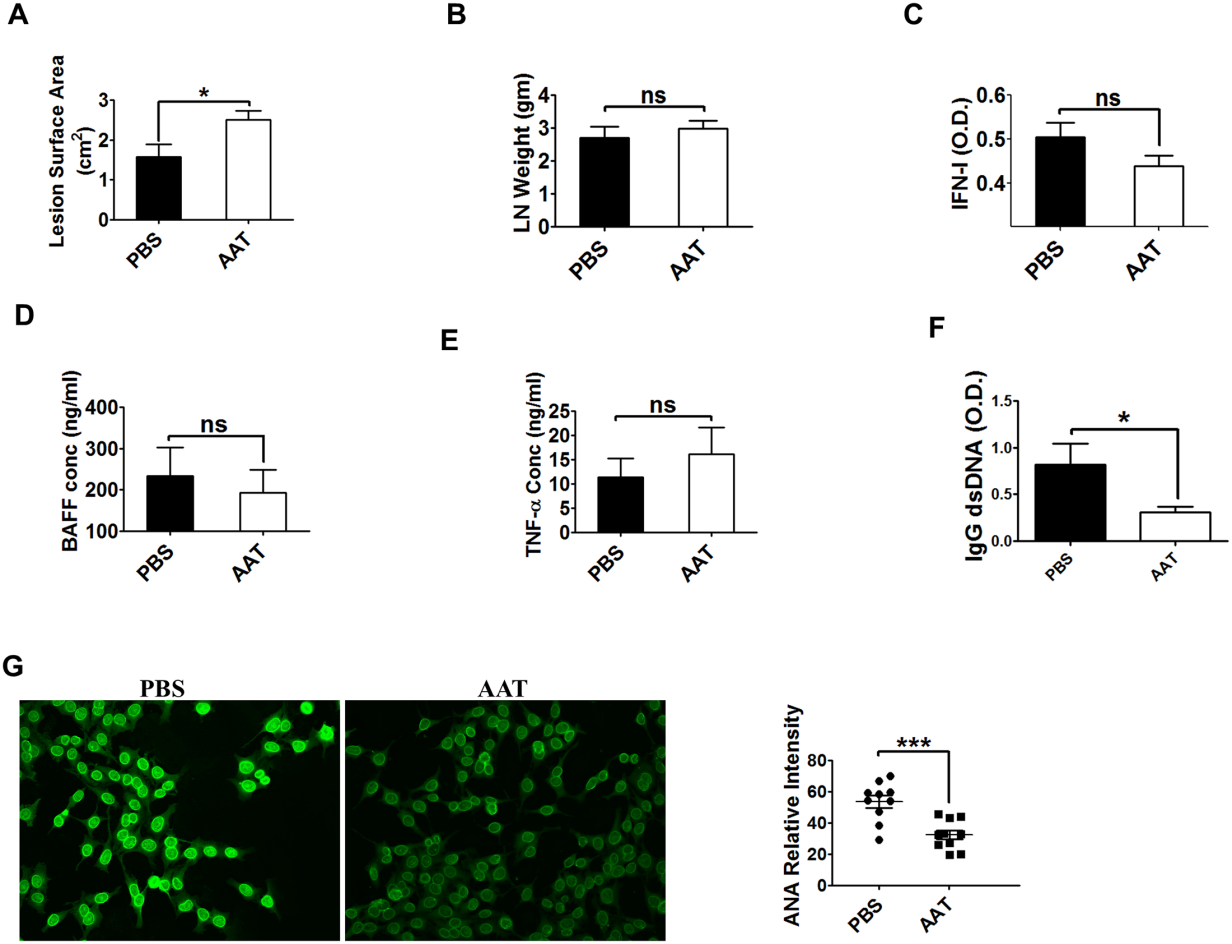
Human AAT inhibited autoantibodies production in MRL/lpr mice. Disease development was evaluated in MRL/lpr mice after 11 weeks of treatment with hAAT or PBS (n = 10 per group). (A) Interscapular lesion surface (cm^2^). (B) Lymph node weight (cervical, brachial, and inguinal). (C) Terminal serum IFN-I, (D) BAFF and (E) TNF-α. (F) Terminal serum anti-dsDNA IgG. (G) Serum ANA staining. Representative images are shown on the left, and FITC relative intensities are graphed on the right. * P<0.05, and*** P<0.001 by Student’s *t*-test.

Human AAT had no protective effect on bodyweight, skin lesion, lymph node weight and serum cytokine levels (Fig 5A–5E). However, serum anti-dsDNA IgG levels were significantly lower in the hAAT treated group than that in the PBS treated control group (Fig 5F). In addition, serum levels of anti-nuclear antibodies (ANAs) were significantly reduced by hAAT treatment (Fig 5G). These results demonstrated that the hAAT treatment is effective in preventing the production of autoantibodies in lupus mice.

**Fig 6:**
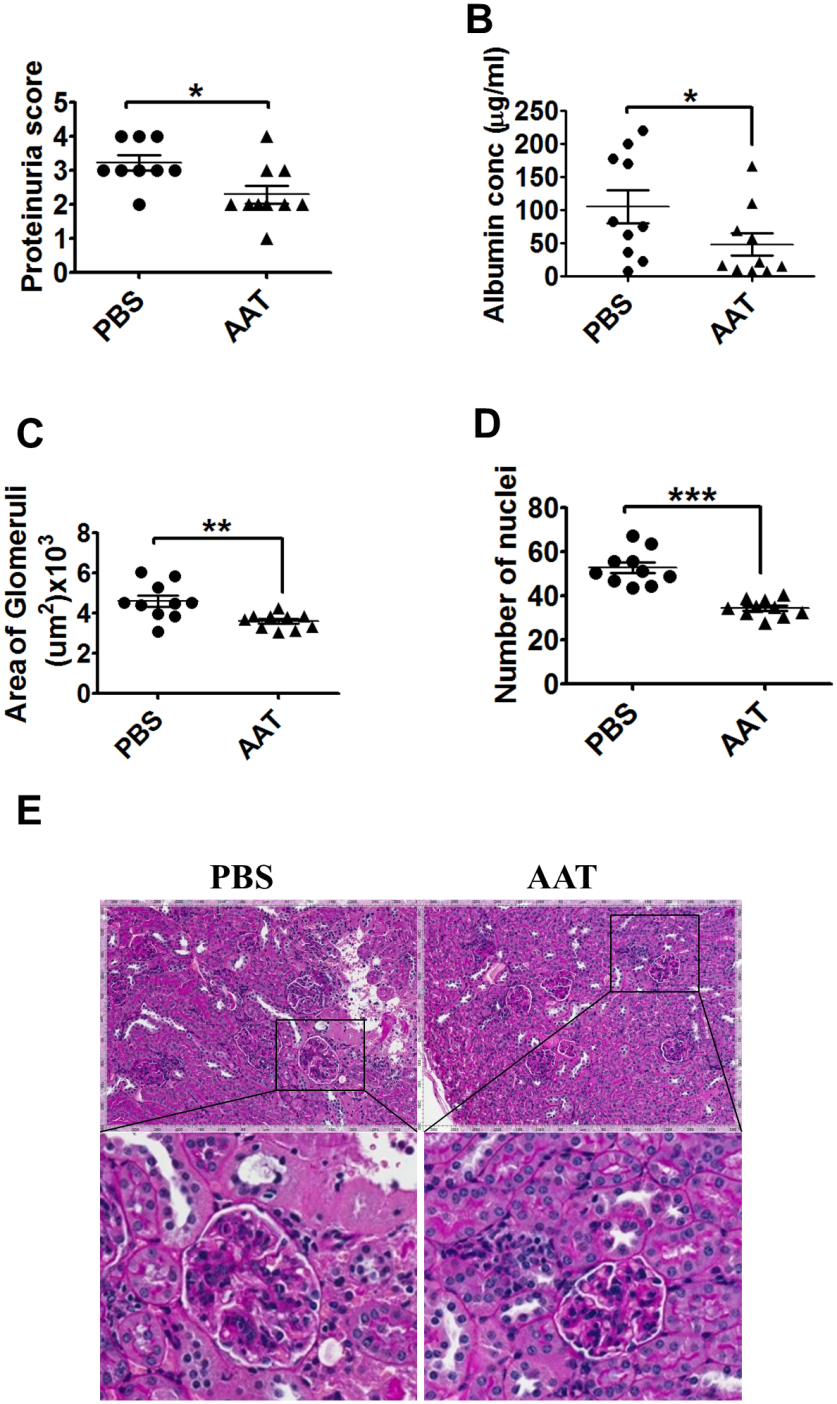
Human AAT treatment attenuated lupus nephritis in MRL/lpr mice. (A) Proteinuria scores after 9 weeks after hAAT treatment (Mann-Whitney test). (B) Albuminuria levels after 11 weeks of hAAT treatment. *P = 0.0727. (C) Average area of glomeruli (um^2^). (D) Average number of nuclei per glomerulus. **P<0.01 and***P<0.001 by student’s *t*-test. (F) Representative PAS-stained kidney sections from control MRL/lpr mice and hAAT treated MRL/lpr mice. All imaged were photographed at the same magnification (20X).

We monitored proteinuria weekly starting at week 4 of treatment. One out of 10 PBS-treated MRL/lpr mice was sacrificed before the 11-week endpoint of the experiment because of high proteinuria, while all of the hAAT-treated mice reached the 11-week endpoint. Human AAT treatment significantly lowered proteinuria levels after 9 weeks of treatment (Fig 6A). Similarly, there was a trend for hAAT treatment lowering urine albumin levels after 11 weeks of treatment (Fig 6B) indicating a renal protective effect of hAAT. Therefore, we next performed comprehensive renal pathological examinations. As shown in Fig 6, the glomeruli in hAAT-treated mice were significantly smaller than that of the control group (Fig 6C and 6E), and the number of glomerular nuclei was significantly lower (Fig 6D and 6E), indicating a reduced mesangial proliferation. These data clearly demonstrated that hAAT treatment attenuated nephritis development in lupus mouse model.

## Discussion

Long-term use of nonsteroidal anti-inflammatory drugs (NSAIDs), antimalarial agents, immunosuppressive drugs, and glucocorticoid for the treatment of SLE may lead to severe side effects. Therefore, the development of new therapeutic options for SLE is needed. In the present study, we showed that hAAT treatment inhibited DC activation and functions, autoantibodies production and importantly attenuated nephropathy in a spontaneous mouse model of lupus. These results strongly indicate that hAAT has a therapeutic potential for the treatment of SLE in humans. Human AAT has been used for the treatment of alpha 1 antitrypsin deficiency (AATD) and tested for the treatment of other diseases including type 1 diabetes [22,27,28,29,44], arthritis [30,31], and GVHD [45]. However, the application of hAAT for the treatment of lupus has not been reported yet. The results from the present study may lead to a novel application for AAT. In addition to its protective effect, AAT has been proven to be a safe drug [46]. Considering that all currently used drugs for lupus have many side effects, the safety feature of AAT may offer a unique venue for the treatment of lupus. Studies have shown that hAAT is a multifunctional protein with proteinase inhibitory, anti-inflammatory and cytoprotective properties. Most of these studies focused on the effect of hAAT on monocytes [26,47], neutrophils [48,49], T cells [27], and pancreatic islet cells [23]. For example, hAAT promoted tolerogenic semi-mature dendritic cells and improved islet transplantation [44,50] and suppressed GVHD [51]. Results from the present study extend the current understanding of hAAT biology and functions in several aspects: 1) hAAT inhibits IFN-I production from DCs. Since IFN-I plays a critical role in initiating innate immunity, the inhibitory effect of hAAT on IFN-I production may be one of the major mechanisms by which hAAT treatment displays therapeutic effects in several disease models including type 1 diabetes, arthritis, stroke and bone loss. 2) hAAT inhibited TLR4 and TLR9-mediated DC stimulation. These data suggest that AAT acts on a common component or factor in the signaling pathways of TLR4 and TLR9. Further dissecting and understanding the effect of hAAT on DC activation and IFN-I productions in normal and lupus models will be critical for the application of hAAT for the treatment of lupus.

To test the functional effects of hAAT on BMDCs, we also examined its effect on the secretion of other inflammatory cytokines. hAAT treatment significantly decreased TNF-α and IL-1β secretion from DCs. It has been suggested that the reduction of co-stimulatory and MHC class II molecules down regulated TNF-α secretion from DCs [52]. A recent study has shown that hAAT can directly interact with cell surface receptors (TNFR1 and TNFR2) and block TNF-α action on the target cells [49]. Consistent with these evidences, we showed that hAAT treatment attenuated the functional responses of DCs and the secretion of pro-inflammatory cytokines.

DCs play a crucial role in the pathogenesis of lupus through IFN-α production upon TLR7-/TLR9 stimulation [53,54]. IFN-α secreted by pDCs promotes auto reactive B cell expansion, differentiate plasma cells to produce autoantibodies and activates myeloid cells and auto reactive T cells [55]. In this study, we demonstrate for the first time that hAAT can inhibit pDCs maturation and secretion of cytokines such as IL-6 and TNF-α upon stimulation with TLR9 ligand. IFN-I was not detected in culture media by the cell assay system, which may be attributed to the low number of differentiated pDCs in bone marrow. Therefore, future studies using pDCs from spleen and lymph nodes may show a better response to hAAT.

It was reported that the chemokine receptor CCR9 was expressed on immature pDCs and that it was down-regulated upon stimulation with either TLR7 or TLR9 [41]. In the present study, we confirmed that CCR9 expressed on BM derived pDCs was down-regulated by stimulation with TLR9 activation and showed that it was significantly up-regulated upon treatment with hAAT.

In this study, we observed that mAAT levels decreased as the disease developed in MRL/lpr mice. The cause for this decrease needs to be further investigated. It is possible that the disease development requires (or consumes) more endogenous mouse AAT for the control of inflammation and tissue damage, yet the diseased mouse cannot produce enough to meet the high demand. Nevertheless, this data supports that additional AAT is needed to control the disease.

IFN-I contributes significantly to renal disease in MRL/lpr murine model of SLE [56]. Our study showed that the treatment of MRL/lpr mice with hAAT every three days for 11 weeks inhibited DCs differentiation and maturation when compared with those from control group. Moreover, hAAT treatment significantly prevented DCs from responding to LPS stimulation compared to the controlled group. Although there was no protective effect on splenomegaly, lymphadenopathy and skin lesion, hAAT treatment significantly reduced serum levels of autoantibodies including anti-dsDNA and ANAs, which are critical for the disease development. More importantly, we also observed the renoprotective effect of hAAT in MRL/lpr mice. We showed that hAAT treatment resulted in significant decreases in proteinuria and urine albumin concentration, as well as nephritis. Our results imply a novel protective function of hAAT, which may play important role in treatment of lupus.

## Conclusion

Our results showed that hAAT treatment significantly inhibited DC activation, autoantibodies production and attenuated renal damage in the lupus mouse model. These results indicate a therapeutic potential of hAAT for the treatment of SLE in humans.

## Supporting Information

S1 Fig Effect of hAAT on cDC differentiation. BM cDCs from B6 mice were generated in vitro in the presence of GM-CSF and IL-4 with or without hAAT (1 mg/ml) for 4 days, followed by LPS 0.5μg/ml or CpG 10μg/ml stimulation for an additional 24 h. Cells were harvested for FACS analysis to detect cDCs. (A) Percent of CD11c+CD11b+, CD11c+ and CD11b+ cells stimulated with or without LPS. (B) Percent of CD11c+CD11b+, CD11c+ and CD11b+ cells stimulated with or without CpG. P values of One-Way-ANOVA using Tukey’s post-hoc test are indicated as * P<0.05, ** P<0.01, and *** P<0.001, n = 3.
(TIF)

S2 Fig Effect of hAAT on pDC differentiation. BM-pDCs from B6 mice were differentiated with or without hAAT (0.6 mg/ml) using Flt3L for 8 days then stimulated with 10μg/ml CpG for an additional 24 h prior to FACS analysis to detect pDCs. (A) Representative FACS plots showing the percentage of PDCA-1+CD11c+ cells. (B) Average percentage of total differentiated pDCs. P values of One-Way-ANOVA using Tukey’s post-hoc test are indicated as *P<0.05, ** P<0.01, and *** P<0.001, n = 3.
(TIF)

S3 Fig Effect of hAAT on pDC maturation. BM-pDCs from B6 mice were differentiated with or without hAAT (1 mg /ml) using Flt3L for 8 days and then stimulated with 10 μg/ml CpG for an additional 24 h prior to FACS analysis. (A and B) Representative FACS histograms showing the MFI (mean fluorescence intensity) of pDCs co-stimulatory molecules CD80 and CD40. (C and D) Statistical analysis for CD80 and CD40 expression on pDCs stimulated with CpG. P values of One-Way-ANOVA using Tukey’s post-hoc test are indicated as *P<0.05, ** P<0.01, and *** P<0.001, n = 3.
(TIF)

S4 Fig Endogenous mouse AAT in culture medium of pDCs from B6 mice. pDCs were differentiated from BM of B6 mice for 8 days using Flt3L followed by 24 h stimulation with or without 10 μg/ml CpG. Medium was collected at day 4 (50% replace) and day 9. Mouse AAT levels in the culture medium were detected by ELISA. (A) Mouse AAT is detectable at day 9 (n = 3). Mean O.D. readings of endogenous mouse AAT levels, m: medium alone (negative control). The dashed line indicates the lower limit of quantification (LLOQ). P values of One-Way-ANOVA using Tukey’s post-hoc test are indicated as * P<0.05, ** P<0.01, and *** P<0.001. (B) CpG stimulation at day 8 does not change endogenous mouse AAT levels. Mean O.D. readings of endogenous mouse AAT levels in pDCs treated with or without CpG for 24 h. Unpaired student’s *t*-test, n = 3.
(TIF)
