# Supplementary figures and images for: Alpha 1 Antitrypsin Inhibits Dendritic Cell Activation and Attenuates Nephritis in a Mouse Model of Lupus
Source: PLoS One. 2016 May 27;11(5):e0156583. doi: 10.1371/journal.pone.0156583 (PMC4883758; doi:10.1371/journal.pone.0156583)

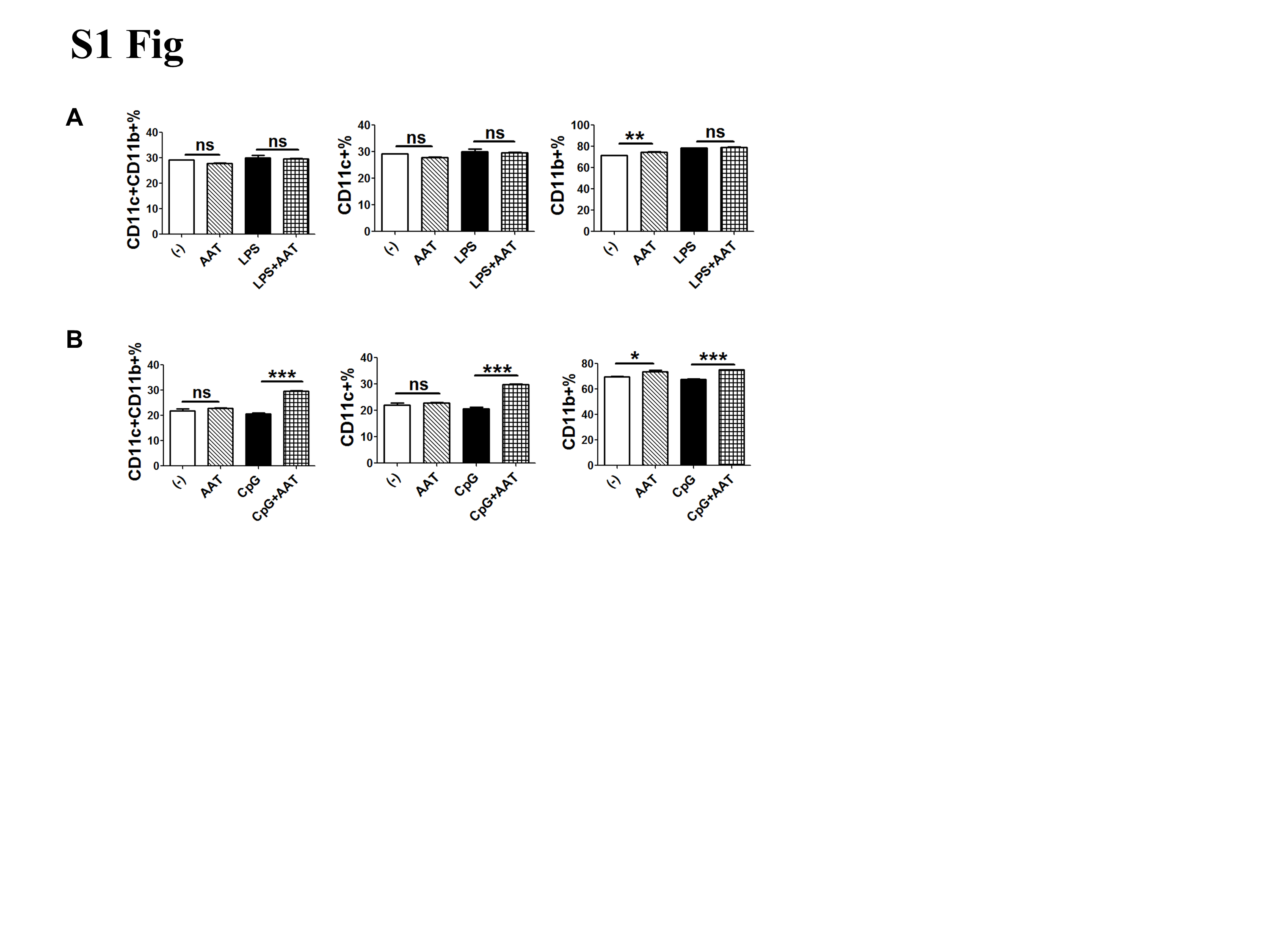

Supplement: S1 Fig — BM cDCs from B6 mice were generated in vitro in the presence of GM-CSF and IL-4 with or without hAAT (1 mg/ml) for 4 days, followed by LPS 0.5μg/ml or CpG 10μg/ml stimulation for an additional 24 h. Cells were harvested for FACS analysis to detect cDCs. (A) Percent of CD11c+CD11b+, CD11c+ and CD11b+ cells stimulated with or without LPS. (B) Percent of CD11c+CD11b+, CD11c+ and CD11b+ cells stimulated with or without CpG. P values of One-Way-ANOVA using Tukey’s post-hoc test are indicated as * P<0.05, ** P<0.01, and *** P<0.001, n = 3. (TIF) [file pone.0156583.s001.tif]

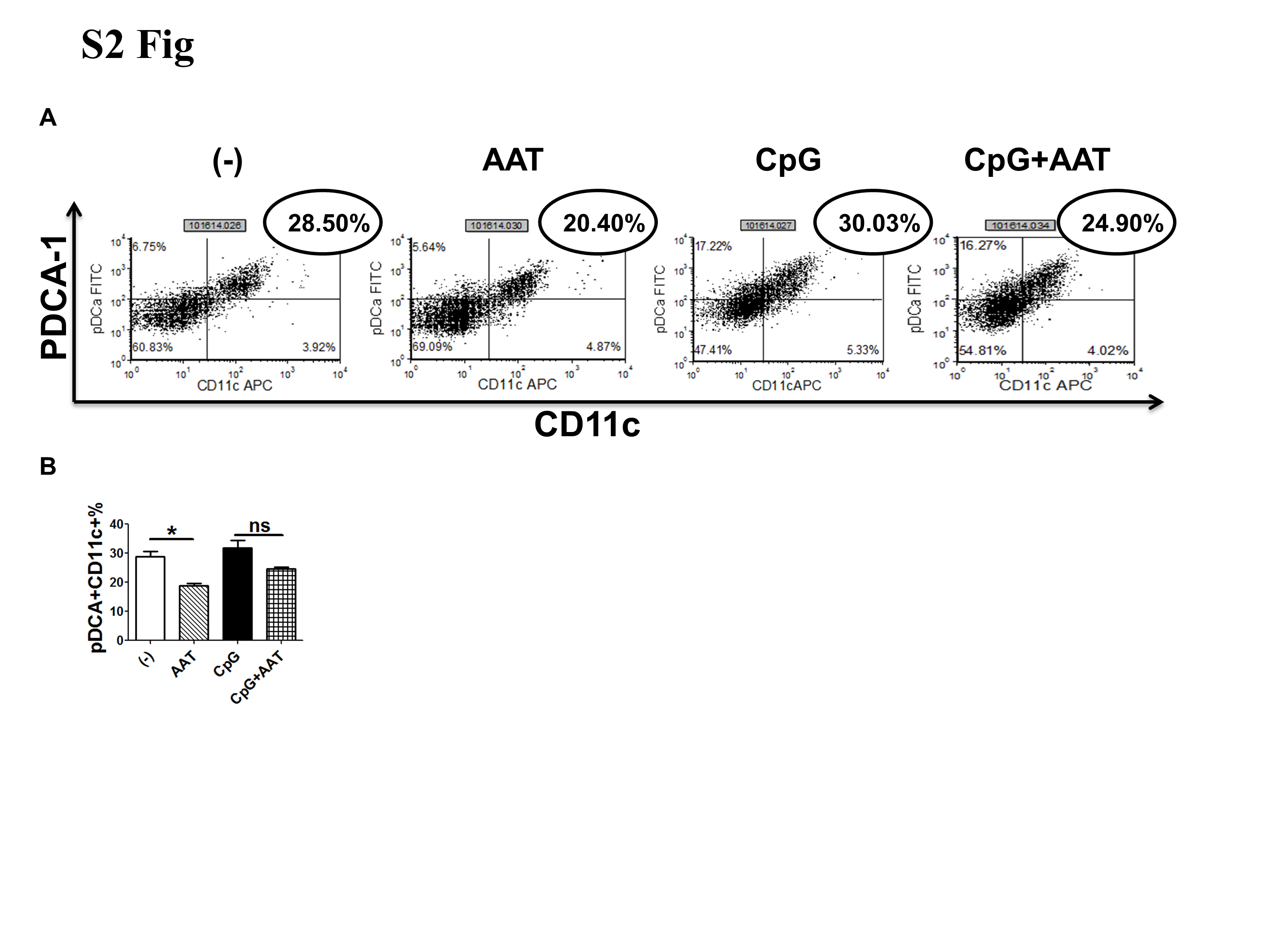

Supplement: S2 Fig — BM-pDCs from B6 mice were differentiated with or without hAAT (0.6 mg/ml) using Flt3L for 8 days then stimulated with 10μg/ml CpG for an additional 24 h prior to FACS analysis to detect pDCs. (A) Representative FACS plots showing the percentage of PDCA-1+CD11c+ cells. (B) Average percentage of total differentiated pDCs. P values of One-Way-ANOVA using Tukey’s post-hoc test are indicated as *P<0.05, ** P<0.01, and *** P<0.001, n = 3. (TIF) [file pone.0156583.s002.tif]

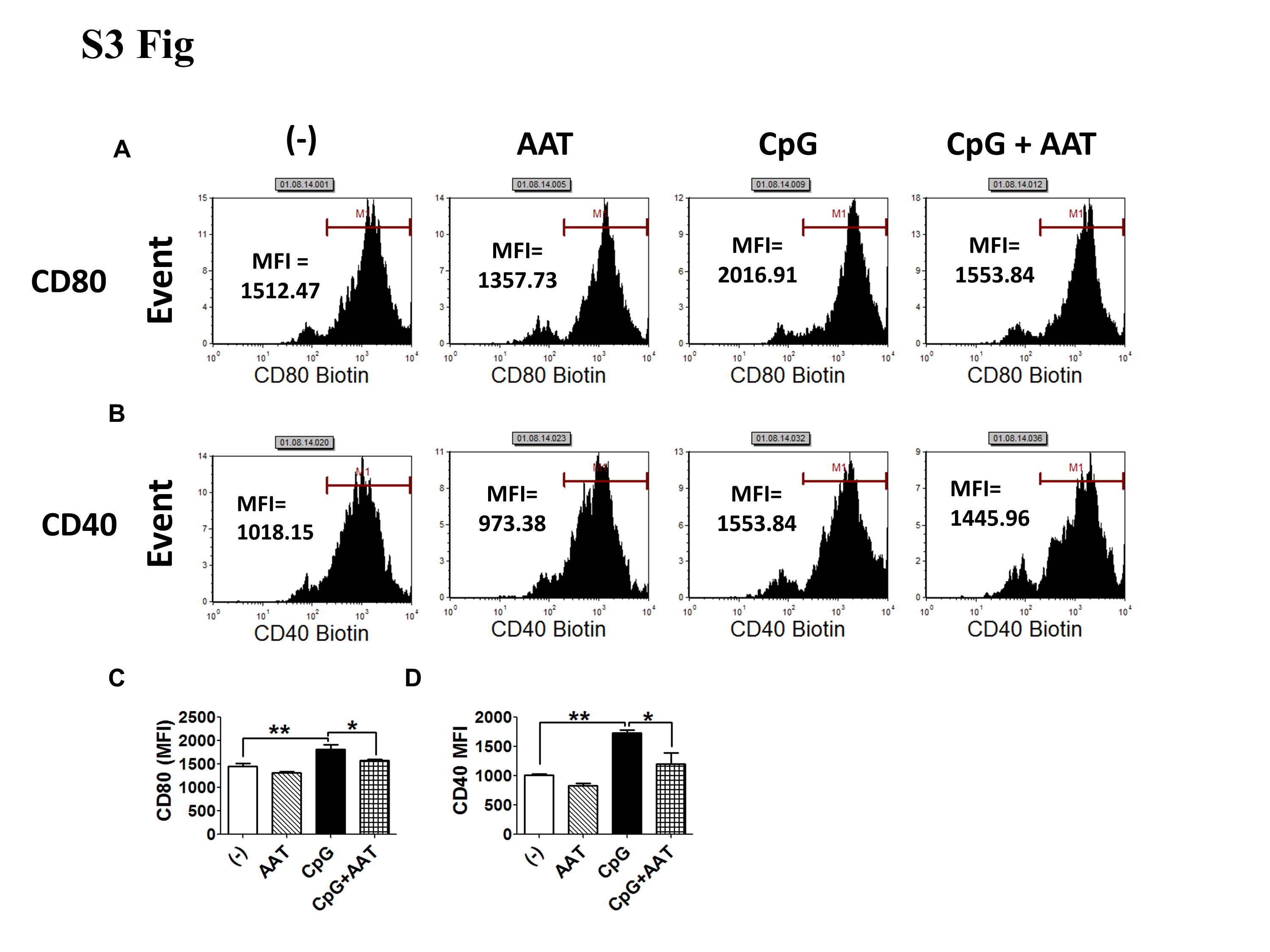

Supplement: S3 Fig — BM-pDCs from B6 mice were differentiated with or without hAAT (1 mg /ml) using Flt3L for 8 days and then stimulated with 10 μg/ml CpG for an additional 24 h prior to FACS analysis. (A and B) Representative FACS histograms showing the MFI (mean fluorescence intensity) of pDCs co-stimulatory molecules CD80 and CD40. (C and D) Statistical analysis for CD80 and CD40 expression on pDCs stimulated with CpG. P values of One-Way-ANOVA using Tukey’s post-hoc test are indicated as *P<0.05, ** P<0.01, and *** P<0.001, n = 3. (TIF) [file pone.0156583.s003.tif]

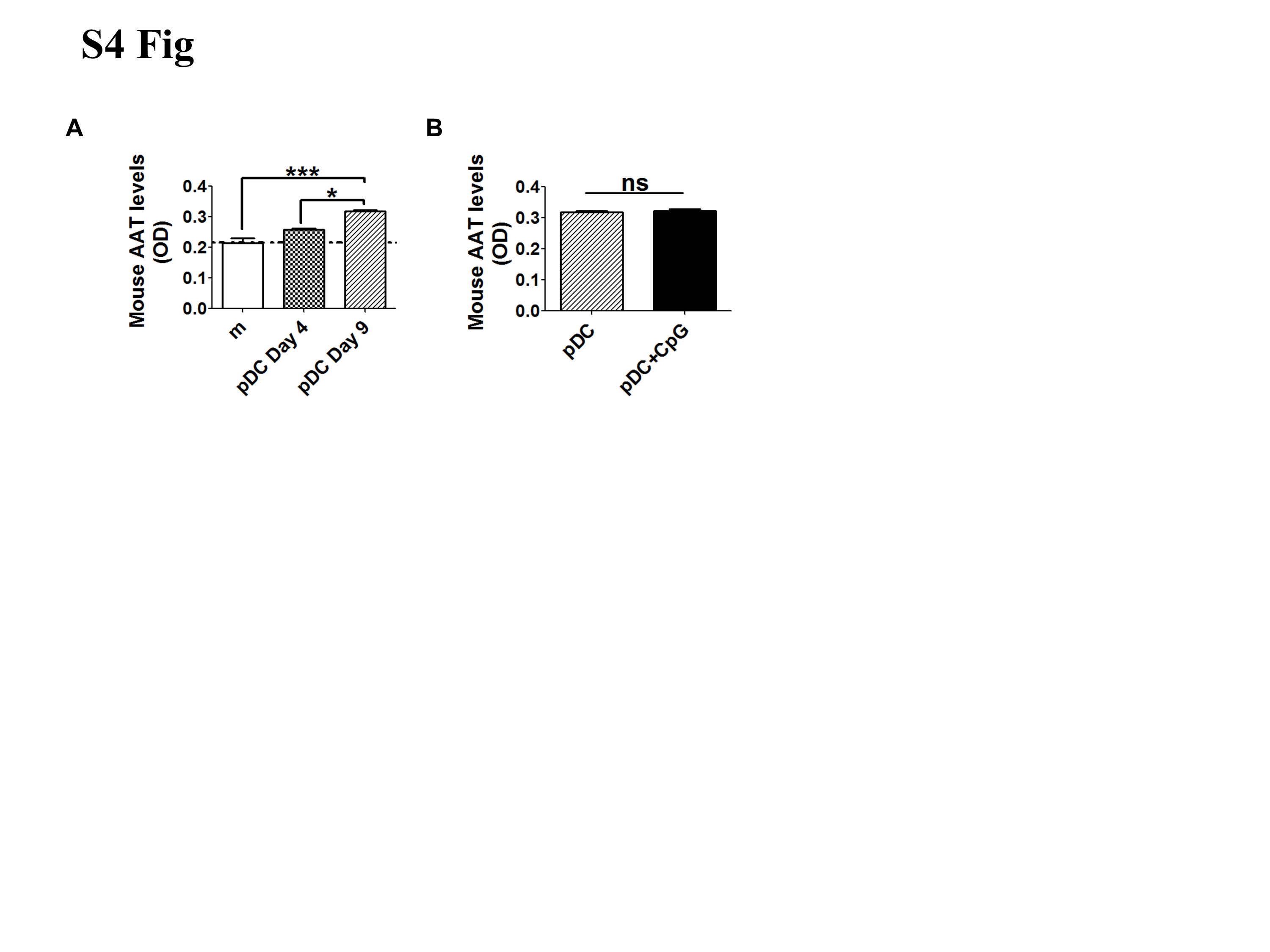

Supplement: S4 Fig — pDCs were differentiated from BM of B6 mice for 8 days using Flt3L followed by 24 h stimulation with or without 10 μg/ml CpG. Medium was collected at day 4 (50% replace) and day 9. Mouse AAT levels in the culture medium were detected by ELISA. (A) Mouse AAT is detectable at day 9 (n = 3). Mean O.D. readings of endogenous mouse AAT levels, m: medium alone (negative control). The dashed line indicates the lower limit of quantification (LLOQ). P values of One-Way-ANOVA using Tukey’s post-hoc test are indicated as * P<0.05, ** P<0.01, and *** P<0.001. (B) CpG stimulation at day 8 does not change endogenous mouse AAT levels. Mean O.D. readings of endogenous mouse AAT levels in pDCs treated with or without CpG for 24 h. Unpaired student’s t-test, n = 3. (TIF) [file pone.0156583.s004.tif]
